# Supplementary material for: Adenosine Induces EBV Lytic Reactivation through ADORA1 in EBV-Associated Gastric Carcinoma
Source: Int J Mol Sci. 2019 Mar 14;20(6):1286. doi: 10.3390/ijms20061286 (PMC6471230; doi:10.3390/ijms20061286)
Supplement: Supplementary file 1 [file ijms-20-01286-s001.pdf]

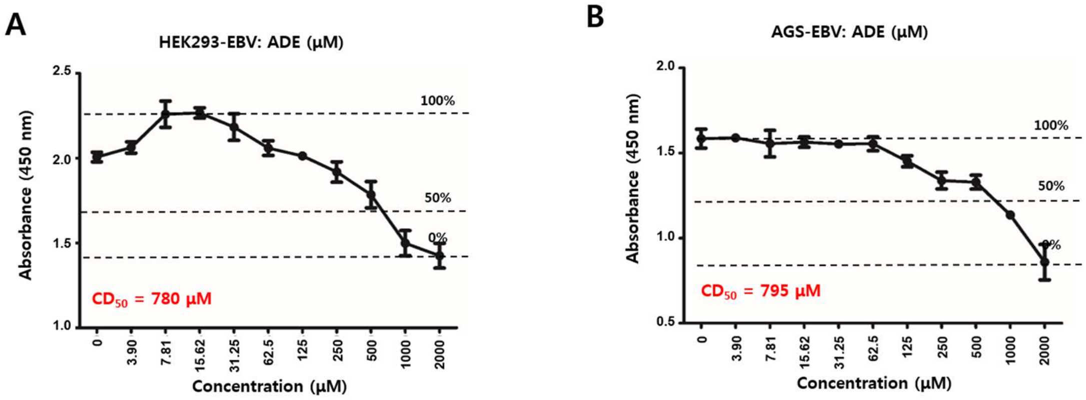

Figure S1. Cytotoxicity for adenosine in HEK293-EBV and AGS-EBV cells. (**A**) Measurement of cytotoxicity of adenosine for 48 h from HEK293-EBV cells; (**B**) Measurement of cytotoxicity of adenosine for 48 h from AGS-EBV cells.

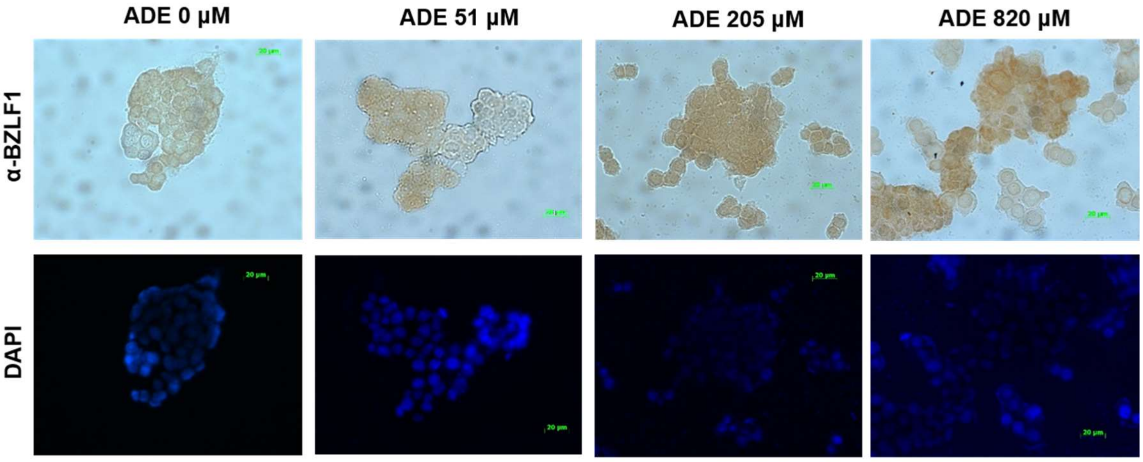

Figure S2. Immunocytochemistry (ICA) assay of SNU719 cells treated with adenosine. Determination of Zta expression in SUN719 cells treated with adenosine. SNU719 cells were methanol-fixed, permeabilized, and probed with mouse anti-Zta antibody and HRP-conjugated goat anti-mouse IgG antibody. HRP was detected using DAB substrate kit. As control, nucleus in SNU719 cells were stained with DAPI.

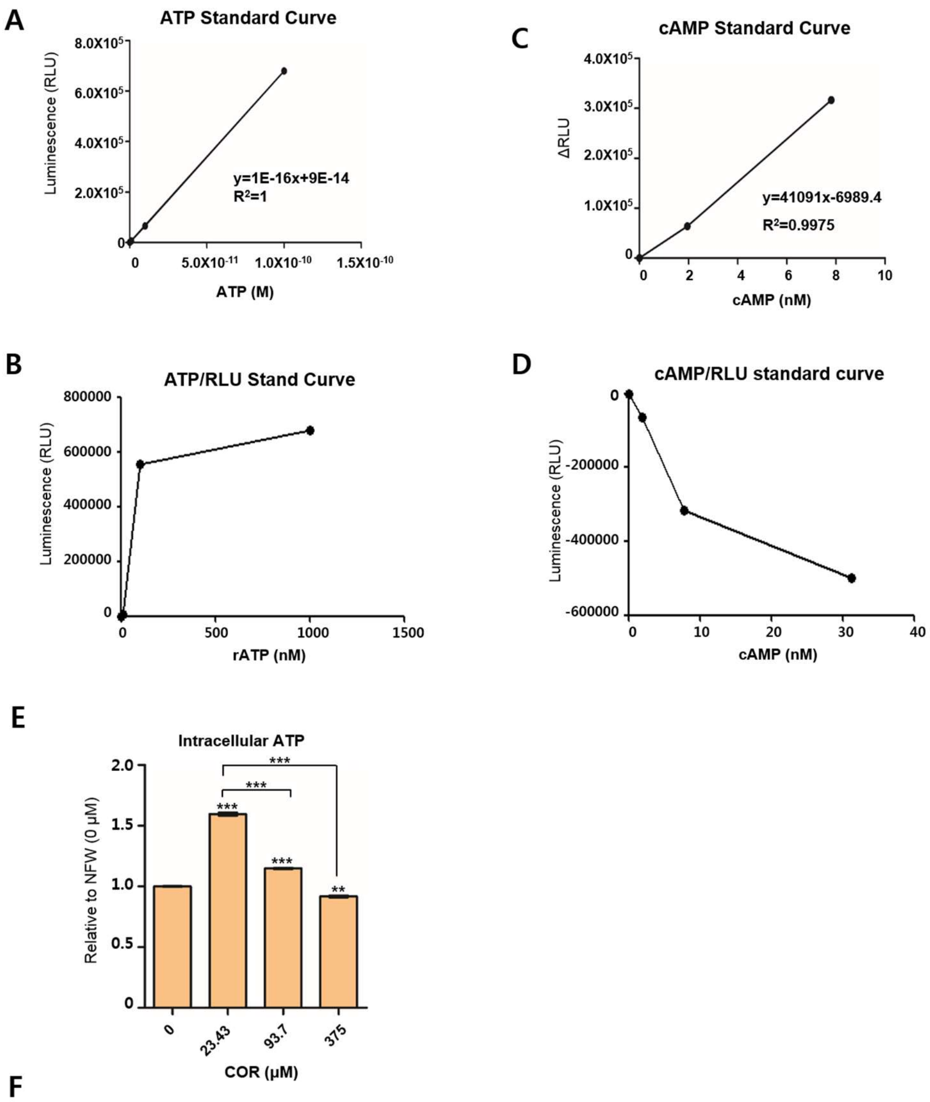

F

ATP standard value

ATP Concentration	Luminoscence			Average
0	642	715	639	665.33
1μM ATP	680262	672731	684761	679251.3
100nM ATP	65985	65424	65377	65595.33
10nM ATP	6383	6435	6634	6484

cAMP standard value

cAMP (nM)	Luminescence			Ave	deltaRLU			Ave	SD
7.8125	181663	184616	183371	183216.7	317915.3	314962.3	316207.3	316361.7	1482.537
1.953125	434541	437539	434815	435631.7	65037.33	62039.33	64763.33	63946.67	1657.471
0	490111	504187	504437	499578.3	9467.333	-4608.67	-4858.67	-1.9E-11	8199.904

Figure S3. Measurement of ATP or cAMP standard curve and ATP induction by cordycepin. (**A**) ATP standard curve for ATP (moles) and Renilla luciferase activity (RLU); (**B**) ATP/RLU standard curve for rATP (nano moles) and Renilla luciferase activity (RLU); (**C**) cAMP standard curve for cAMP (moles) and Renilla luciferase activity (RLU); (**D**) Measurement of net cAMP induction by adenosine in SNU719 cells. Net cAMP levels were represented as RLU directly relative to the adenosine concentration; (**E**) Measurement of ATP induction by cordycepin in SNU719 cells. SNU719 cells were treated with 23.43, 93.70, and 375.00 μM of cordycepin for 48 h. ATP levels are represented relative to the NFW control (0 μM); (**F**) Luminescence of ATP and cAMP standard. ADE, COR and NFW = adenosine, cordycepin and nuclease-free water, respectively. ** $p < 0.01$, *** $p < 0.001$ (Student's *t*-test).

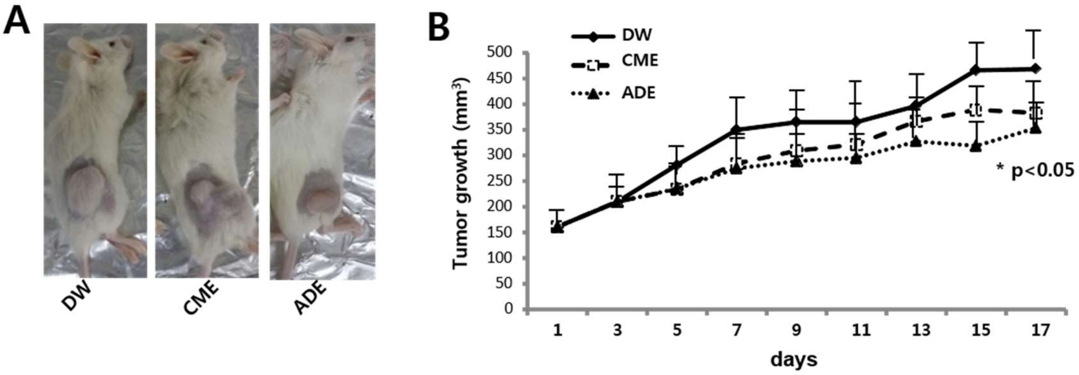

Figure S4. Inhibition of SNU719 development in xenograft mouse by CME and adenosine. (**A**) Representative image of a NOD/SCID mouse bearing MKN1-EBV cells-derived tumors; (**B**) Determination of the anti-tumor effects of CME and adenosine on the development of MKN1-EBV cells-derived tumors in NOD/SCID mice. Each experimental groups were served drinking water, CME (100 mg/kg/day) and ADE (30 mg/kg/day). CME, ADE, and DW = *Cordyceps militaris* extracts, adenosine, and drinking water, respectively.
